# Supplementary material for: Adding function to the genome of African Salmonella Typhimurium ST313 strain D23580
Source: PLoS Biol. 2019 Jan 15;17(1):e3000059. doi: 10.1371/journal.pbio.3000059 (PMC6333337; doi:10.1371/journal.pbio.3000059)
Supplement: S1 Table — ST, sequence type. (PDF) [file pbio.3000059.s001.pdf]

| Phenotype | ST313 lineage 2 | ST19 | References |
|---|---|---|---|
| ***In vitro* assays** | | | |
| Human serum killing resistance. | Increased[a]. High expression of PgtE due to a SNP in the gene promoter region. | Decreased[b]. | [1] |
| Ability to ferment melibiose. | Negative. | Positive. | [2] |
| Utilization of the carbon sources L-tartaric acid and dihydroxyacetone. | Decreased[a]. *ttdA* is a pseudogene. | Increased[b]. | [3] |
| Preference for the alternative carbon sources meso-tartaric acid and tricarballylic acid. | Increased[a]. *ttdA* is a pseudogene. | Decreased[b]. | [3] |
| Ability to ferment inositol. | Positive. | Variable. | [2] |
| Ability to ferment sugars to pyruvate via the butylene glycol pathway (Voges-Proskauer test). | Positive. | Negative. | [2] |
| Ability to use citrate as sole carbon source. | Positive. | Negative. | [2] |
| Utilization of purine and pyrimidine nucleotides as sources of phosphorus. | Decreased[a]. | Increased[b]. | [3] |
| Swimming motility on soft agar. | Decreased[a] [4,5]. Decreased[a] FliC protein level [4]. Conflicting results showed D23580 more motile than SL1344 [2]. | Increased[b]. | [2,4,5] |
| Resistance to acid stress (pH 3.5). | Increased[a]. | Decreased[b]. | [2] |
| Amount of complement required for antibody-mediated bactericidal activity. | Increased[a]. | Decreased[b]. | [6] |
| Stationary-phase catalase production. | Decreased[a]. E117G KatE mutation [7]. | Increased[b]. | [2,7] |
| Ability to form RDAR (red, dry and rough) colonies on Congo red agar. | Intermediate phenotype [8]. Negative due to a premature stop codon in *bcsG* at position 247 [7]. | Increased[b]. | [7,8] |
| Biofilm production in a continuous flow system. | Decreased[a]. | Increased[b]. | [8] |
| Survival after desiccation. | Decreased[a]. | Increased[b]. | [8] |
| Pellicle formation. | Negative. | Positive. | [8] |
| Biofilm formation in a static system (crystal violet assay). | Decreased[a]. | Increased[b]. | [8] |
| Genome degradation. | 77 pseudogenes in D23580 and large deletions. | 33 pseudogenes in LT2. | [3,9] |

| | | | |
|---|---|---|---|
| Acetylation of the rhamnose in the O-antigen of the LPS. | Yes.<br>Modification carried out by GtrC[BTP1]. | No (in LT2). | [10,11] |
| ~$10^9$ BTP1 virus particles per mL in supernatants of non-induced, stationary-phase cultures. | Yes.<br>Presence of the BTP1 prophage. | No. | [12] |
| **Cellular infection models** | | | |
| Invasiveness of epithelial cells. | Decreased[a] [3,13,14].<br>Lower[a] mRNA levels of *sopE2* in SPI-1-inducing conditions [13].<br>However, D23580 was more invasive than 14028 [7].<br>Heterogeneity of results in [15]. | Increased[b]. | [3,7,13–15] |
| Replication/survival in epithelial cells and in a 3-dimensional co-culture infection model of colonic epithelial cells and macrophages. | Increased[a]. | Decreased[b]. | [14] |
| Invasiveness of a 3-dimensional co-culture infection model of colonic epithelial cells and macrophages. | Decreased[a]. | Increased[b]. | [14] |
| Uptake in macrophages. | Increased[a]. | Decreased[b]. | [4,7] |
| Survival/replication in macrophages. | Increased[a] [4,16].<br>No differences[a] (D23580 versus 14028) [7].<br>Heterogeneity of results in [15]. | Decreased[b]. | [4,7,15,16] |
| Cytotoxicity in macrophages. | Increased[a] (D23580 versus 14028). | Decreased[b]. | [7] |
| Induction of caspase-1-dependent macrophage death and IL-1β release. | Decreased[a]. | Increased[b]. | [13] |
| Apoptosis and inflammatory response in macrophages. | Decreased[a]. | Increased[b].<br>Only in part due to an increased[b] FliC protein levels. | [4] |
| NLRC4 inflammasome activation in macrophages. | Decreased[a].<br>Lower[a] levels of *fliC* mRNA in SPI-1-inducing conditions (confirmed at the protein level). | Increased[b]. | [13] |
| Resistance to killing by macrophages | Increased[a]. | Decreased[b]. | [4] |
| Presence of *bstA* (formerly *st313-td*), contributes to uptake and intracellular survival within macrophages, and virulence in mice. | Present in the BTP1 prophage. | Absent. | [16] |
| **Animal infection models** | | | |

| | | | |
|---|---|---|---|
| Fluid accumulation in bovine ligated ileal loop model and rhesus macaque ligated ileal loop assay. | Decreased[a] in bovine ligated ileal loop model [3]. However, no differences[a] in the rhesus macaque ligated ileal loop assay (D23580 versus 14028) [7]. | Increased[b] in bovine ligated ileal loop model [3]. However, no differences[b] in the rhesus macaque ligated ileal loop assay (D23580 versus 14028) [7]. | [3,7] |
| Induction of inflammatory response in the caecum of streptomycin pre-treated mice. | Decreased[a] in C57BL/6 mice [3], but no differences[a] in pathology in CBA/J mice [7] and CD-1 mice [5]. | Increased[b]. | [3,5,7] |
| Colonic colonization in streptomycin pre-treated mice. | Increased[a] in CBA/J mice. | Decreased[b]. | [7] |
| Colonization in mesenteric lymph nodes (MLN) in streptomycin pre-treated mice. | Increased[a] in CBA/J mice [7] and C57BL/6 mice [17]. Hyperdissemination from the gut via infected migratory DCs. Pseudogenization of *sseI* [17]. | Decreased[b]. | [7,17] |
| Levels of bacteraemia in BALB/c mice. | Increased[a]. | Decreased[b]. | [5] |
| Ability to colonize the spleen in BALB/c mice. | Increased[a]. | Decreased[b]. | [2] |
| Clinical signs in rhesus macaques. | No significant pathology. | Varying degrees of pathology in the liver, ileum, colon and MLN. | [5] |
| Invasive infection of spleen and liver in a chicken infection model. | Increased[a]. | Decreased[b]. | [18] |

[a] ST313 lineage 2 compared with ST19 isolates.

[b] ST19 compared with ST313 lineage 2 isolates.

## Supporting References


1. Hammarlöf DL, Kröger C, Owen SV, Canals R, Lacharme-Lora L, Wenner N, et al. Role of a single noncoding nucleotide in the evolution of an epidemic African clade of Salmonella. Proc Natl Acad Sci U S A. 2018;115: E2614–E2623. doi:10.1073/pnas.1714718115

2. Yang J, Barrila J, Roland KL, Kilbourne J, Ott CM, Forsyth RJ, et al. Characterization of the Invasive, Multidrug Resistant Non-typhoidal Salmonella Strain D23580 in a Murine Model of Infection. PLoS Negl Trop Dis. 2015;9: e0003839. doi:10.1371/journal.pntd.0003839

3. Okoro CK, Barquist L, Connor TR, Harris SR, Clare S, Stevens MP, et al. Signatures of Adaptation in Human Invasive Salmonella Typhimurium ST313 Populations from Sub-Saharan Africa. PLoS Negl Trop Dis. 2015;9. doi:10.1371/journal.pntd.0003611

4. Ramachandran G, Perkins DJ, Schmidlein PJ, Tulapurkar ME, Tennant SM. Invasive Salmonella Typhimurium ST313 with Naturally Attenuated Flagellin Elicits Reduced Inflammation and Replicates within Macrophages. PLoS Negl Trop Dis. 2015;9: e3394. doi:10.1371/journal.pntd.0003394

5. Ramachandran G, Panda A, Higginson EE, Ateh E, Lipsky MM, Sen S, et al. Virulence of invasive Salmonella Typhimurium ST313 in animal models of infection. PLoS Negl Trop Dis. 2017;11: e0005697. doi:10.1371/journal.pntd.0005697

6. Goh YS, MacLennan CA. Invasive African nontyphoidal Salmonella requires high levels of complement for cell-free antibody-dependent killing. J Immunol Methods. 2013;387: 121–129. doi:10.1016/j.jim.2012.10.005

7. Singletary LA, Karlinsey JE, Libby SJ, Mooney JP, Lokken KL, Tsolis RM, et al. Loss of Multicellular Behavior in Epidemic African Nontyphoidal Salmonella enterica Serovar Typhimurium ST313 Strain D23580. mBio. 2016;7. doi:10.1128/mBio.02265-15

8. Ramachandran G, Aheto K, Shirtliff ME, Tennant SM. Poor biofilm-forming ability and long-term survival of invasive Salmonella Typhimurium ST313. Pathog Dis. 2016;74. doi:10.1093/femspd/ftw049

9. Kingsley RA, Msefula CL, Thomson NR, Kariuki S, Holt KE, Gordon MA, et al. Epidemic multiple drug resistant Salmonella Typhimurium causing invasive disease in sub-Saharan Africa have a distinct genotype. Genome Res. 2009;19: 2279–2287. doi:10.1101/gr.091017.109

10. Kintz E, Davies MR, Hammarlöf DL, Canals R, Hinton JCD, van der Woude MW. A BTP1 prophage gene present in invasive non-typhoidal Salmonella determines composition and length of the O-antigen of the lipopolysaccharide. Mol Microbiol. 2015;96: 263–275. doi:10.1111/mmi.12933

11. Micoli F, Ravenscroft N, Cescutti P, Stefanetti G, Londero S, Rondini S, et al. Structural analysis of O-polysaccharide chains extracted from different Salmonella Typhimurium strains. Carbohydr Res. 2014;385: 1–8. doi:10.1016/j.carres.2013.12.003

12. Owen SV, Wenner N, Canals R, Makumi A, Hammarlöf DL, Gordon MA, et al. Characterization of the Prophage Repertoire of African Salmonella Typhimurium ST313 Reveals High Levels of Spontaneous Induction of Novel Phage BTP1. Front Microbiol. 2017;8. doi:10.3389/fmicb.2017.00235

13. Carden S, Okoro C, Dougan G, Monack D. Non-typhoidal Salmonella Typhimurium ST313 isolates that cause bacteremia in humans stimulate less inflammasome activation than ST19 isolates associated with gastroenteritis. Pathog Dis. 2015;73. doi:10.1093/femspd/ftu023

14. Barrila J, Yang J, Crabbé A, Sarker SF, Liu Y, Ott CM, et al. Three-dimensional organotypic co-culture model of intestinal epithelial cells and macrophages to study Salmonella enterica colonization patterns. NPJ Microgravity. 2017;3. doi:10.1038/s41526-017-0011-2

15. Almeida F, Seribelli AA, da Silva P, Medeiros MIC, dos Prazeres Rodrigues D, Moreira CG, et al. Multilocus sequence typing of Salmonella Typhimurium reveals the presence of the highly invasive ST313 in Brazil. Infect Genet Evol. 2017;51: 41–44. doi:10.1016/j.meegid.2017.03.009

16. Herrero-Fresno A, Wallrodt I, Leekitcharoenphon P, Olsen JE, Aarestrup FM, Hendriksen RS. The Role of the st313-td Gene in Virulence of Salmonella Typhimurium ST313. PLOS ONE. 2014;9: e84566. doi:10.1371/journal.pone.0084566

17. Carden SE, Walker GT, Honeycutt J, Lugo K, Pham T, Jacobson A, et al. Pseudogenization of the Secreted Effector Gene sseI Confers Rapid Systemic Dissemination of S. Typhimurium ST313 within Migratory Dendritic Cells. Cell Host Microbe. 2017;21: 182–194. doi:10.1016/j.chom.2017.01.009

18. Parsons BN, Humphrey S, Salisbury AM, Mikoleit J, Hinton JCD, Gordon MA, et al. Invasive Non-Typhoidal Salmonella Typhimurium ST313 Are Not Host-Restricted and Have an Invasive Phenotype in Experimentally Infected Chickens. PLoS Negl Trop Dis. 2013;7: e2487. doi:10.1371/journal.pntd.0002487
