# Supplementary material for: Adding function to the genome of African Salmonella Typhimurium ST313 strain D23580
Source: PLoS Biol. 2019 Jan 15;17(1):e3000059. doi: 10.1371/journal.pbio.3000059 (PMC6333337; doi:10.1371/journal.pbio.3000059)
Supplement: S9 Table — (PDF) [file pbio.3000059.s009.pdf]

Resource	Source	Identifier
**Bacterial Strains**		
Salmonella Typhimurium 4/74 (ST19)	[1]	JH3676
Salmonella Typhimurium D23580 (ST313 lineage 2)	[2]	JH3621
Salmonella Typhimurium 14028 (ST19)	L. Bossi	MA5958 (JH3829)
Salmonella Typhimurium D25248 (ST313 lineage 1)	[2]	IC24T
Salmonella Typhimurium A130 (ST313 lineage 1)	[2]	IC24S
Salmonella Typhimurium U3 (UK-ST313 strain)	[3]	IC25G
Salmonella Typhimurium U2 (UK-ST313 strain)	[3]	IC25F
Salmonella Typhimurium D37712 (ST313 lineage 2)	[4]	IC24O
Salmonella Typhimurium D23580 *flhA*$^{4/74}$	This study	JH4303
Salmonella Typhimurium 4/74 *melB*$^-$ (contains the two D23580 *melB* SNPs)	This study	JH4304
Salmonella Typhimurium 4/74 *melR*$^-$ (contains the D23580 *melR* SNP)	This study	JH4305
Salmonella Typhimurium 4/74 *melB*$^-$ *melR*$^-$ (contains the two D23580 *melB* SNPs and the D23580 *melR* SNP)	This study	JH4306
Salmonella Typhimurium D23580 *melB*$^+$ (contains the two 4/74 *melB* SNPs)	This study	JH4307
Salmonella Typhimurium D23580 *melR*$^+$ (contains the 4/74 *melR* SNP)	This study	JH4308
Salmonella Typhimurium D23580 *melB*$^+$ *melR*$^+$ (contains the two 4/74 *melB* SNPs and the 4/74 *melR* SNP)	This study	JH4309
Salmonella Typhimurium D23580 *ΔcysS*^{chr}::*frt*	This study	JH4298
Salmonella Typhimurium D23580 *ΔcysS*^{pBT1}::*frt*	This study	JH4299
Salmonella Typhimurium D23580 ΔpBT1	This study	JH4300
E. coli S17-1 λ*pir* (*pro thi hsdR recA* chromosome::RP4-2 Tc::Mu Km::Tn7/λ*pir*; Tp^R, Sm^R)	[5]	JH3867
**Plasmids**		
frt-aph-frt cassette template plasmid; Km^R	[6]	pKD4
λ Red recombination plasmid, temperature-inducible; Tc^R	[7]	pSIM5-*tet*
FLP recombinase expression plasmid; Tc^R	[8]	pCP20-TcR
pKD4 derivative with an I-SceI site cloned upstream of *aph*; Km^R	[9]	pKD4-I-SceI
Plasmid for *m*-toluate–inducible expression of the I-SceI enzyme; Gm^R	[10]	pSW-2
Suicide plasmid; Km^R	[10]	pEMG

Supporting References

1. Rankin JD, Taylor RJ. The estimation of doses of Salmonella typhimurium suitable for the experimental production of disease in calves. Vet Rec. 1966;78: 706–707.

2. Kingsley RA, Msefula CL, Thomson NR, Kariuki S, Holt KE, Gordon MA, et al. Epidemic multiple drug resistant Salmonella Typhimurium causing invasive disease in sub-Saharan Africa have a distinct genotype. Genome Res. 2009;19: 2279–2287. doi:10.1101/gr.091017.109

3. Ashton PM, Owen SV, Kaindama L, Rowe WPM, Lane CR, Larkin L, et al. Public health surveillance in the UK revolutionises our understanding of the invasive Salmonella Typhimurium epidemic in Africa. Genome Med. 2017;9. doi:10.1186/s13073-017-0480-7

4. Msefula CL, Kingsley RA, Gordon MA, Molyneux E, Molyneux ME, MacLennan CA, et al. Genotypic Homogeneity of Multidrug Resistant S. Typhimurium Infecting Distinct Adult and Childhood Susceptibility Groups in Blantyre, Malawi. PLOS ONE. 2012;7: e42085. doi:10.1371/journal.pone.0042085

5. Simon R, Priefer U, Pühler A. A Broad Host Range Mobilization System for In Vivo Genetic Engineering: Transposon Mutagenesis in Gram Negative Bacteria. Nat Biotechnol. 1983;1: 784–791. doi:10.1038/nbt1183-784

6. Datsenko KA, Wanner BL. One-step inactivation of chromosomal genes in Escherichia coli K-12 using PCR products. Proc Natl Acad Sci U S A. 2000;97: 6640–6645.

7. Koskiniemi S, Pränting M, Gullberg E, Näsvall J, Andersson DI. Activation of cryptic aminoglycoside resistance in Salmonella enterica. Mol Microbiol. 2011;80: 1464–1478. doi:10.1111/j.1365-2958.2011.07657.x

8. Kintz E, Davies MR, Hammarlöf DL, Canals R, Hinton JCD, van der Woude MW. A BTP1 prophage gene present in invasive non-typhoidal Salmonella determines composition and length of the O-antigen of the lipopolysaccharide. Mol Microbiol. 2015;96: 263–275. doi:10.1111/mmi.12933

9. Owen SV, Wenner N, Canals R, Makumi A, Hammarlöf DL, Gordon MA, et al. Characterization of the Prophage Repertoire of African Salmonella Typhimurium ST313 Reveals High Levels of Spontaneous Induction of Novel Phage BTP1. Front Microbiol. 2017;8. doi:10.3389/fmicb.2017.00235

10. Martínez-García E, Lorenzo V de. Engineering multiple genomic deletions in Gram-negative bacteria: analysis of the multi-resistant antibiotic profile of Pseudomonas putida KT2440. Environ Microbiol. 2011;13: 2702–2716. doi:10.1111/j.1462-2920.2011.02538.x
