# Supplementary material for: Adding function to the genome of African Salmonella Typhimurium ST313 strain D23580
Source: PLoS Biol. 2019 Jan 15;17(1):e3000059. doi: 10.1371/journal.pbio.3000059 (PMC6333337; doi:10.1371/journal.pbio.3000059)
Supplement: S10 Table — RNA-seq, RNA sequencing. (PDF) [file pbio.3000059.s010.pdf]

Condition	Description[a]
EEP (early exponential phase)	Growth in Lennox broth at 37ºC, 220 rpm (in a water bath), to $OD_{600\ nm}$ 0.1.
MEP (middle exponential phase)	Growth in Lennox broth at 37ºC, 220 rpm (in a water bath), to $OD_{600\ nm}$ 0.3.
LEP (late exponential phase)	Growth in Lennox broth at 37ºC, 220 rpm (in a water bath), to $OD_{600\ nm}$ 1.
ESP (early stationary phase)	Growth in Lennox broth at 37ºC, 220 rpm (in a water bath), to $OD_{600\ nm}$ 2.
LSP (late stationary phase)	Growth in Lennox broth at 37ºC, 220 rpm (in a water bath), to $OD_{600\ nm}$ 2, followed by a further 6 h growth in the same incubation conditions.
25ºC	Growth in Lennox broth at 25ºC, 220 rpm (in a water bath), to $OD_{600\ nm}$ 0.3.
NaCl shock	Growth in Lennox broth at 37ºC, 220 rpm (in a water bath), to $OD_{600\ nm}$ 0.3, then addition of NaCl to a final concentration of 0.3 M for 10 min in the same incubation conditions.
Bile shock	Growth in Lennox broth at 37ºC, 220 rpm (in a water bath), to $OD_{600\ nm}$ 0.3, then addition of bile to a final concentration of 3% for 10 min in the same incubation conditions.
Low Fe^{2+} shock	Growth in Lennox broth at 37ºC, 220 rpm (in a water bath), to $OD_{600\ nm}$ 0.3, then addition of 2,2'-dipyridyl to a final concentration of 0.2 mM for 10 min in the same incubation conditions.
Anaerobic shock	Growth in Lennox broth at 37ºC, 220 rpm (in a water bath), to $OD_{600\ nm}$ 0.3, then transfer of 15 ml into a 15 ml Falcon tube and incubated statically at 37ºC for 30 min.
Anaerobic growth	Growth in Lennox broth in a completely filled and closed 50 ml Falcon tube, incubated statically at 37ºC, to $OD_{600\ nm}$ 0.3.
Oxygen shock	Growth in Lennox broth in a completely filled and closed 50 ml Falcon tube, incubated statically at 37ºC, to $OD_{600\ nm}$ 0.3, then transfer into a baffled flask for 15 min at 37ºC, 250 rpm (in a water bath).
NonSPI2 (SPI-2-noninducing)	Growth in PCN medium (pH 7.4, 25 mM P_i) at 37ºC, 220 rpm (in a water bath), to $OD_{600\ nm}$ 0.3.
InSPI2 (SPI-2-inducing)	Growth in PCN medium (pH 5.8, 0.4 mM P_i) at 37ºC, 220 rpm (in a water bath), to $OD_{600\ nm}$ 0.3.
Peroxide shock (InSPI2)	Growth in PCN medium (pH 5.8, 0.4 mM P_i) at 37ºC, 220 rpm (in a water bath), to $OD_{600\ nm}$ 0.3, then addition of H_2O_2 to a final concentration of 1 mM for 12 min in the same incubation conditions.
Nitric oxide shock (InSPI2)	Growth in PCN medium (pH 5.8, 0.4 mM P_i) at 37ºC, 220 rpm (in a water bath), to $OD_{600\ nm}$ 0.3, then addition of spermine NONOate to a final concentration of 250 µM for 20 min in the same incubation conditions.
Macrophage	Bacteria from the intra-macrophage (RAW264.7) environment were recovered after 8 h post-infection[b].

[a] Precise details of the growth conditions, including the components of the phosphate carbon nitrogen (PCN)-related minimal media and the type of water bath that was used, have been published previously in Kröger and colleagues [1].

[b] A detailed protocol for this condition was described in Srikumar and colleagues [2].

Supporting References

1. Kröger C, Colgan A, Srikumar S, Händler K, Sivasankaran SK, Hammarlöf DL, et al. An Infection-Relevant Transcriptomic Compendium for Salmonella enterica Serovar Typhimurium. Cell Host Microbe. 2013;14: 683–695. doi:10.1016/j.chom.2013.11.010

2. Srikumar S, Kröger C, Hébrard M, Colgan A, Owen SV, Sivasankaran SK, et al. RNA-seq Brings New Insights to the Intra-Macrophage Transcriptome of Salmonella Typhimurium. PLOS Pathog. 2015;11: e1005262. doi:10.1371/journal.ppat.1005262
