# Supplementary material for: Adding function to the genome of African Salmonella Typhimurium ST313 strain D23580
Source: PLoS Biol. 2019 Jan 15;17(1):e3000059. doi: 10.1371/journal.pbio.3000059 (PMC6333337; doi:10.1371/journal.pbio.3000059)
Supplement: S11 Table — (PDF) [file pbio.3000059.s011.pdf]

**Oligonucleotide sequence (5’→3’)**	**Identifier**
AACCGAGGTAACTGGCTTG	Fw-pBT2-1
TGTCAGCACGTTCAGGAAG	Rv-pBT2-1
AAGCGTTCCTCACTGTATCG	Fw-pBT2-2
TGTCATTCCGCTGTTATGG	Rv-pBT2-2
CCTCCGTAACAGGTTTTCG	Fw-pBT3-3
AATCCGGCTTGATACGATG	Rv-pBT3-3
CAAAACCTACCGCCTGAAT	Fw-pBT3-1
CATCTTGTCCGGTTTCGAC	Rv-pBT3-4
TGAAAGATTCGCAGCAGAG	Fw-pBT3-4
CAGGTGATATTCCGCTTCC	Rv-pBT3-2
TACGTCTTTCCAGATGCAGG	DH-21
GAATTAATACGACTCACTATATGAATCGTATAACAGCCCCG	DH-22
GTTGAATCGGCATTCTGCTC	DH-25
GAATTAATACGACTCACTATAAGAGCTCCTTACCCGTCA	DH-26
GTATTTGTGTCAATGACAGAG	DH-29
GAATTAATACGACTCACTATAGGTGGTCTGTGGTATACATA	DH-30
ATAGAACTGCTCTTCAATACC	DH-33
GAATTAATACGACTCACTATAGATGATTACAAATCACTTGGG	DH-34
ACCAATCAGACCGGCATTAAGATCGGCGTCAATCGCCATTTGCTTACC GGGCATACCATCCAGCACAAAGC	flhA-474SNP
GCGGCGGTAAAAAGTAAAA	Fw-flhA
ATGCCCGGTAAGCAAATGG	Rv-flhA-474SNP
GGAATTCCAGACAGTCAATGGCTTCG	melR-EcoRI-F
CGGGATCCATGGCGATTTTCAGTTTGC	melR-BamHI-R
GGAATTCTCTCTTGCCTGTTGGGAAT	melB-EcoRI-F
CGGGATCCTACTGGCACAGCAGAGCAT	melB-BamHI-R
ATCATACGGAGATTATTACCCACACACGTCTATACGGAATCTTCGTGTA GGCTGGAGCTGCTTC	NW_206
AAAAAACAGGCCGTTCTATGCAAATAGAGCGGCCTGTAAAAGCGCCAT ATGAATATCCTCCTTAG	NW_207
GGCTCTATTGATTAAGTAAACGAACTACTATCAGGAATGTCATAAGTGT AGGCTGGAGCTGCTTC	NW_210
AGCTAAACAGGCTTACCTATAGCGATAACGGTACTATTCAATTAACATA TGAATATCCTCCTTAG	NW_211
AGCAACCAGCAACTGCACGATATAGATCTGGATAGCTACTTATGACATT TCGAACCCCAGAGTCC	NW_163
AATTACCACAGCCGGGTGTAGAACATTGAATATCACTCATAGTCACTAG GGATAACAGGGTAATC	NW_164
