# Supplementary material for: Adding function to the genome of African Salmonella Typhimurium ST313 strain D23580
Source: PLoS Biol. 2019 Jan 15;17(1):e3000059. doi: 10.1371/journal.pbio.3000059 (PMC6333337; doi:10.1371/journal.pbio.3000059)
Supplement: S1 Text — (PDF) [file pbio.3000059.s012.pdf]

## Reannotation of *S.* Typhimurium D23580

The two indels confirmed by resequencing were used to update published annotated coding genes. The locations of the transcriptional start sites (TSS) defined in [1] were also updated following the introduction of the two indels. New gene annotations were based on comparative genetic analysis versus 4/74, and RNA-seq data, and were confirmed by the presence of putative start and stop codons. Comparative genetic analysis involved identifying genes annotated in 4/74 and absent in the D23580 annotation. The sequence of those genes was searched in D23580 and annotated if they met the criteria of ≥95% identity between strains and chromosomal synteny. Some new genes in D23580 were annotated based on the presence of an open reading frame (ORF) and transcription in the RNA-seq data. Different length for the same gene in the two strains has an effect on TPM values and could generate misinterpretation of RNA-seq data. Hence, the locations of the 'start' and 'end' of some coding genes were changed based on the locations in the 4/74 annotation when no nucleotide sequence differences were found between strains.

Noncoding sRNAs from 4/74 [2,3] were identified in D23580 by BLAST [4] with >95% identity at the nucleotide level. Annotation of the eight novel noncoding sRNAs in the BTP1 region was based on RNA-seq data, with TSS already defined in [1]. The presence of promoter and terminator regions for these sRNAs was confirmed by sequence analysis.

The updated annotation of D23580 coding genes and sRNAs is presented in S2 Table.

## Annotation of pBT1 plasmid

The annotation of pBT1 was based on a draft version provided by Robert A. Kingsley, and improved using RAST [5–7].

## SNP and indel analysis between strains

Snippy v3.1 with "--ctgs" option was used to identify single-nucleotide polymorphisms and small insertions and deletions between 4/74 and D23580 strains. Visual inspection of simulated short read coverage was used to identify regions of low similarity in prophage and bordering regions; these were excluded from the obtained variant calls as false positives. SnpEff v4.3t [8] with custom-made database was used to annotate variant types (noncoding, synonymous, missense, gain/loss of stop, and indel) for the identified variants.

**Orthology analysis between *S*. Typhimurium 4/74 and D23580 strains**

A total of 4413 orthologous genes and sRNAs between strains D23580 and 4/74 were determined using Roary [9] and a 95% sequence identity for the BLASTP component. Three BTP5-encoded genes (*gpD*, *STMMW_32051*, *STMMW_32101*) and one BTP1-encoded gene (*STMMW_03941*) located within prophage regions were removed from the ortholog gene list. Six genes in the pSLT-BT plasmid showed homology with genes in the 4/74-specific pRSF1010$^{4/74}$ and were excluded: *repA*, *repC*, *sulII*, *strA*, *strB*, and *tnpB*. Lastly, 272 noncoding sRNAs were added as orthologs. There were a final of 4,675 orthologous genes and noncoding sRNAs between the two strains. The IsrB-1 ncRNA was further removed for the comparative transcriptomic analysis due to duplication in D23580.

**Orthology analysis between *S.* Typhimurium D23580, LT2, 14028, 4/74, and UK-1 strains**

Roary v3.12.0 [9] was used with the paralogue splitting option to identify the orthologous protein-coding genes in five *S.* Typhimurium strains: D23580, LT2, 14028, 4/74, and UK-1. NCBI GFF3 annotations from LT2, 14028, 4/74, and UK-1 (accession: AE006468.2 and AE006471.2, CP001363.1 and CP001362, CP002487.1 and CP002488.1, and CP002614.1 and CP002615.1, respectively), and our D23580 custom annotation were pre-processed, with only protein coding genes retained, and with locus tags used as protein IDs. It should be noted that LT2 gene feature annotation differed from CDS features, and CDS coordinates were used instead. A default of 95% BLASTP protein sequence similarity cutoff was used to identify orthologous genes. The pan-genomic analysis identified 5,938 protein-coding genes, 4,255 of which were present in all five strains (“core” genes). Orthologues are listed in S2 Table.

**Northern blotting**

DIG-labelled riboprobes were generated by PCR and T7 *in vitro* transcription using the DIG Northern Starter Kit (Roche). Primer sequences are included in S11 Table and were designed as previously described [2]. The oligonucleotides used for each specific noncoding sRNA were: for STnc6000, DH21 and DH22; for STnc6020, DH25 and DH26; for STnc6040, DH29 and DH30; and for STnc6060, DH33 and DH34. 5 or 10 μg of total RNA were loaded onto 7% polyacrylamide, 8.3 M Urea in 1x TBE gels. RNA was subsequently transferred to a positively charged nylon membrane and UV-crosslinked. Hybridization and detection procedures were performed according to manufacturer’s instructions (DIG

Northern Starter Kit, Roche). Bands were visualised using an Image Quant LAS 4000 imager (GE Healthcare Life Sciences).

### Swimming motility assay

Strains were grown for 16 h in Lennox broth, 37ºC 220 rpm, and bacterial cultures were diluted to O.D.$_{600nm}$ 2 in Lennox broth. 3 µL of the dilutions were spotted onto the surface of Lennox 0.3% agar plates. The diameter of migration represented swimming motility, and was measured after 5 h at 37ºC.

### Bone marrow-derived macrophages (BMDM) cytotoxicity assay

Bone marrow-derived C57BL/6 macrophages were differentiated for 5 days in DMEM (Invitrogen) with 10% FBS (Thermo Fisher Scientific), 20% MCSF (L929 cell supernatant) and 10 mM HEPES (Invitrogen). One day before infection, macrophages were seeded into 96-well plates at densities of 50,000 cells *per* well in DMEM with 10% FBS, 10% MSCF and 10 mM HEPES, and stimulated with 100 ng/mL LPS (Invitrogen) for 16 h. Overnight cultures of *Salmonella* strains were subcultured 1:50 and incubated statically for 3.5 h at 37ºC. Bacteria were then resuspended in DMEM with 10% FBS, 10% MSCF and 10 mM HEPES at a multiplicity of infection (M.O.I.) of 20. Medium was removed from macrophages and replaced with the medium containing *Salmonella*. Macrophages were spun for 5 min to synchronize infection and incubated for 1 h. Cell death was quantified by measuring the amount of lactate dehydrogenase (LDH) released into the supernatant compared to an uninfected total lysis control at 1 h post-infection. LDH was measured using the Cytotox96 non-radioactive cytotoxicity kit (Promega) as previously described [10]. One-way ANOVA and Tukey’s multiple comparison test were used for statistical analysis.

## Supporting Results

### Identified pBT1 homologues

Plasmids in databases that showed homology with pBT1 included *Salmonella* sp. 14 plasmid p14-95A (99% identity over 97% coverage, accession: JQ418537) and *Salmonella* sp. 40 plasmid p40-95A (98% identity over 94% coverage, accession: JQ418539), both plasmids isolated in the USA; and an unnamed plasmid from *S. enterica* subsp. *salamae* serovar 55:k:z39 str. 1315K (99% identity over 92% coverage, accession: CP022141), from Madagascar. Two other unnamed plasmids sharing high similarity were present in *S. enterica* subsp. *enterica* serovar Onderstepoort str. SA20060086 (99%

identity over 97% coverage, accession: CP022036) and *S. enterica* subsp. *diarizonae* serovar 65:c:z str. SA20044251 (98% identity over 92% coverage, accession: CP022136).

## Supporting References


1. Hammarlöf DL, Kröger C, Owen SV, Canals R, Lacharme-Lora L, Wenner N, et al. Role of a single noncoding nucleotide in the evolution of an epidemic African clade of Salmonella. Proc Natl Acad Sci U S A. 2018;115: E2614–E2623. doi:10.1073/pnas.1714718115
2. Kröger C, Colgan A, Srikumar S, Händler K, Sivasankaran SK, Hammarlöf DL, et al. An Infection-Relevant Transcriptomic Compendium for Salmonella enterica Serovar Typhimurium. Cell Host Microbe. 2013;14: 683–695. doi:10.1016/j.chom.2013.11.010
3. Kröger C, Dillon SC, Cameron ADS, Papenfort K, Sivasankaran SK, Hokamp K, et al. The transcriptional landscape and small RNAs of Salmonella enterica serovar Typhimurium. Proc Natl Acad Sci. 2012;109: E1277–E1286. doi:10.1073/pnas.1201061109
4. Altschul SF, Gish W, Miller W, Myers EW, Lipman DJ. Basic local alignment search tool. J Mol Biol. 1990;215: 403–410. doi:10.1016/S0022-2836(05)80360-2
5. Aziz RK, Bartels D, Best AA, DeJongh M, Disz T, Edwards RA, et al. The RAST Server: Rapid Annotations using Subsystems Technology. BMC Genomics. 2008;9: 75. doi:10.1186/1471-2164-9-75
6. Brettin T, Davis JJ, Disz T, Edwards RA, Gerdes S, Olsen GJ, et al. RASTtk: A modular and extensible implementation of the RAST algorithm for building custom annotation pipelines and annotating batches of genomes. Sci Rep. 2015;5. doi:10.1038/srep08365
7. Overbeek R, Olson R, Pusch GD, Olsen GJ, Davis JJ, Disz T, et al. The SEED and the Rapid Annotation of microbial genomes using Subsystems Technology (RAST). Nucleic Acids Res. 2014;42: D206–D214. doi:10.1093/nar/gkt1226
8. Cingolani P, Platts A, Wang LL, Coon M, Nguyen T, Wang L, et al. A program for annotating and predicting the effects of single nucleotide polymorphisms, SnpEff. Fly (Austin). 2012;6: 80–92. doi:10.4161/fly.19695
9. Page AJ, Cummins CA, Hunt M, Wong VK, Reuter S, Holden MTG, et al. Roary: rapid large-scale prokaryote pan genome analysis. Bioinformatics. 2015;31: 3691–3693. doi:10.1093/bioinformatics/btv421
10. Carden S, Okoro C, Dougan G, Monack D. Non-typhoidal Salmonella Typhimurium ST313 isolates that cause bacteremia in humans stimulate less inflammasome activation than ST19 isolates associated with gastroenteritis. Pathog Dis. 2015;73. doi:10.1093/femspd/ftu023
