# Supplementary figures and images for: Adding function to the genome of African Salmonella Typhimurium ST313 strain D23580
Source: PLoS Biol. 2019 Jan 15;17(1):e3000059. doi: 10.1371/journal.pbio.3000059 (PMC6333337; doi:10.1371/journal.pbio.3000059)

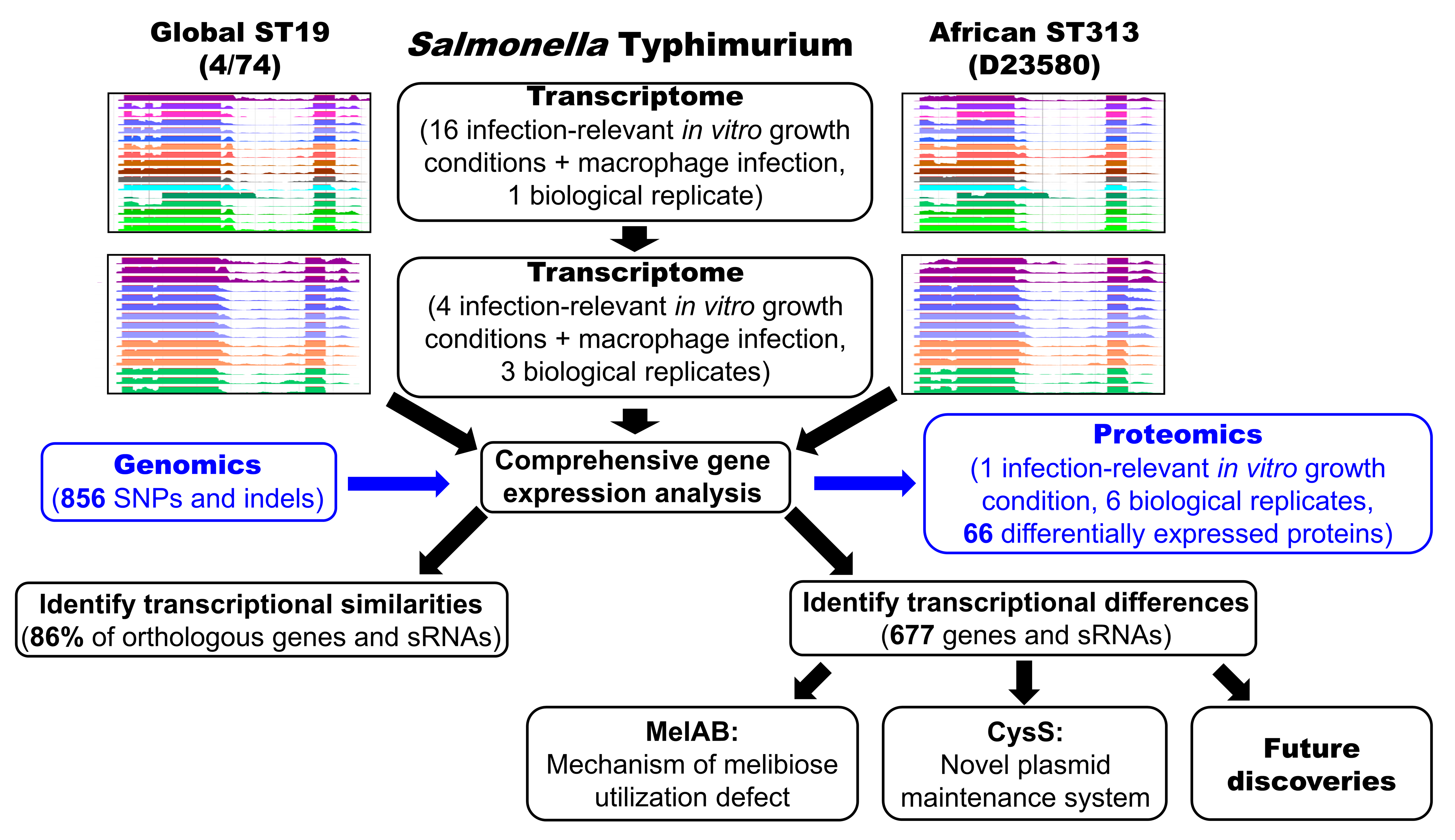

Supplement: S1 Fig — RNA-seq, RNA sequencing. (TIF) [file pbio.3000059.s013.tif]

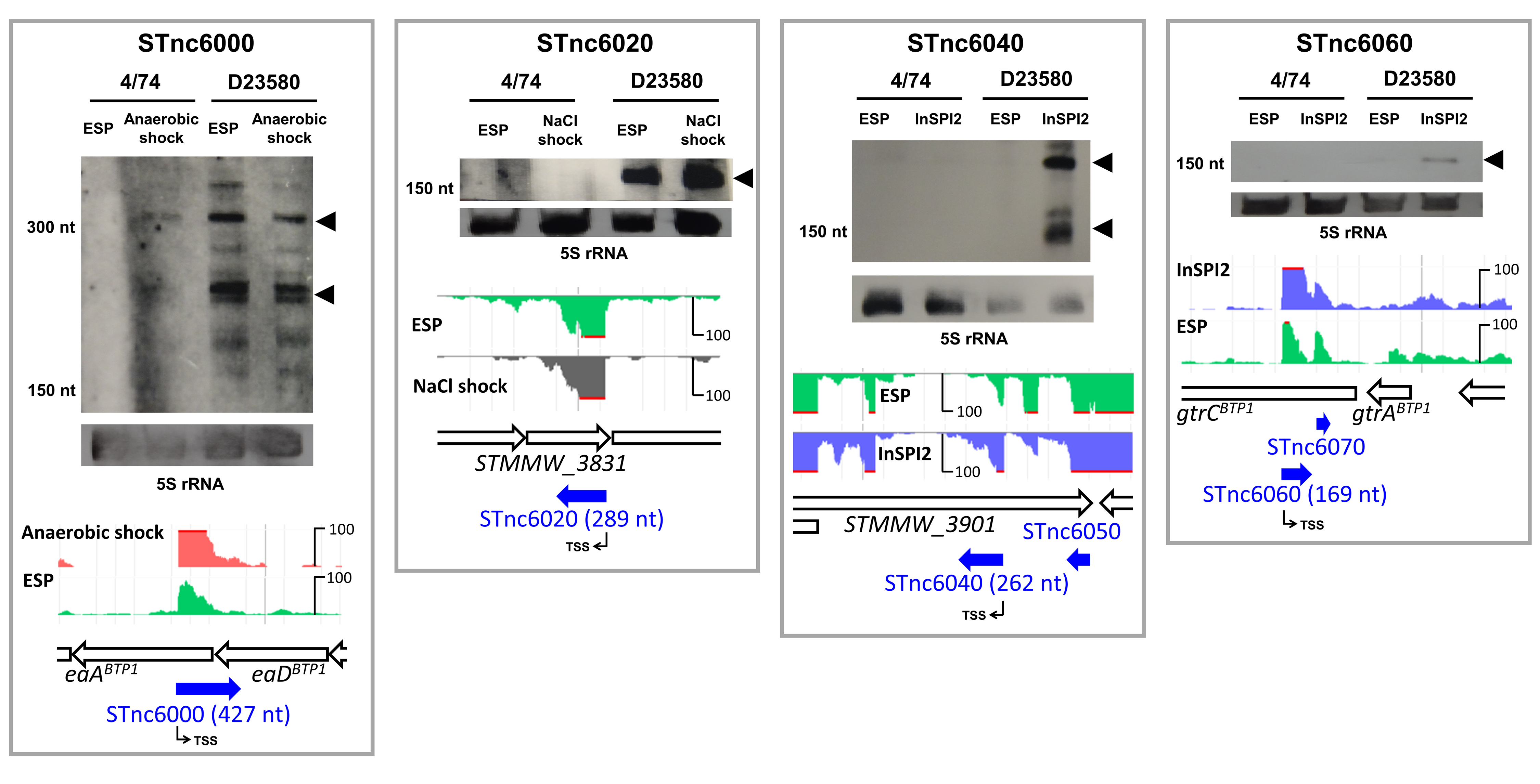

Supplement: S2 Fig — Northern blots confirming the existence of novel sRNAs annotated in the BTP1 prophage region (S1 Text). For every individual sRNA, a northern blot and mapped reads in the same conditions are shown. The arrowheads indicate the most prominent bands. 5S rRNA was used as a loading control. Estimated length of the sRNAs is in brackets and was based on RNA-seq data and sequence analysis. TSSs of the individual sRNAs are indicated at the bottom. RNA-seq, RNA sequencing; sRNA, small RNA; TSS, transcriptional start site; 5S rRNA, ribosomal 5S RNA. (TIF) [file pbio.3000059.s014.tif]

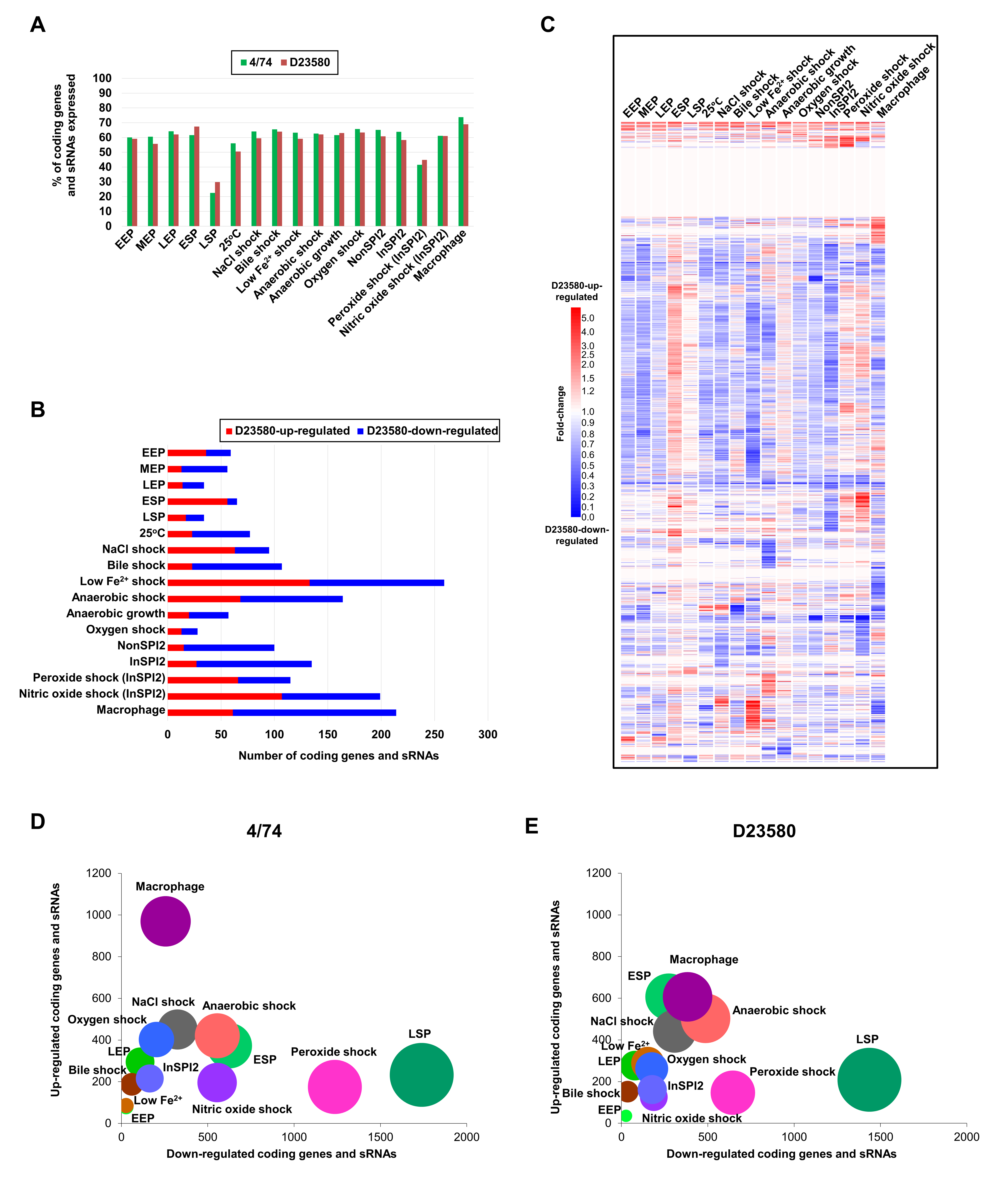

Supplement: S3 Fig — (A) Percentage of expressed genes (TPM >10) for each individual strain (S5 Table). (B) Number of coding genes and sRNAs differentially expressed (fold-change ≥3) for each of the 17 infection-relevant conditions (S5 Table). (C) Heat map of the cluster analysis of all orthologous coding genes and sRNAs between the two strains obtained using GeneSpring GX7.3 (Agilent). The TPM for each coding gene and sRNA in each condition in D23580 was divided by the TPM value for the same gene/sRNA and condition in 4/74. TPM values ≤10 (representing nonexpressed genes or sRNAs) were set to 10 before calculating fold-changes (S5 Table). (D) Bubble chart for 4/74 representing up-regulated coding genes and sRNAs versus down-regulated. The following comparisons based on TPM values were obtained for each specific condition: MEP, LEP, ESP, and LSP were compared to EEP; NaCl shock, bile shock, low Fe2+ shock, and anaerobic shock were compared to MEP; oxygen shock was compared to anaerobic growth; peroxide shock and nitric oxide shocks were compared to InSPI2; InSPI2 was compared to NonSPI2; and macrophage was compared to ESP. (E) Bubble chart for D23580. EEP, early exponential phase; ESP, early stationary phase; InSPI2, SPI-2-inducing; LEP, late exponential phase; LSP, late stationary phase; MEP, middle exponential phase; NonSPI2, SPI-2-noninducing; SPI, Salmonella pathogenicity island; sRNA, small RNA; TPM, transcripts per million. (TIF) [file pbio.3000059.s015.tif]

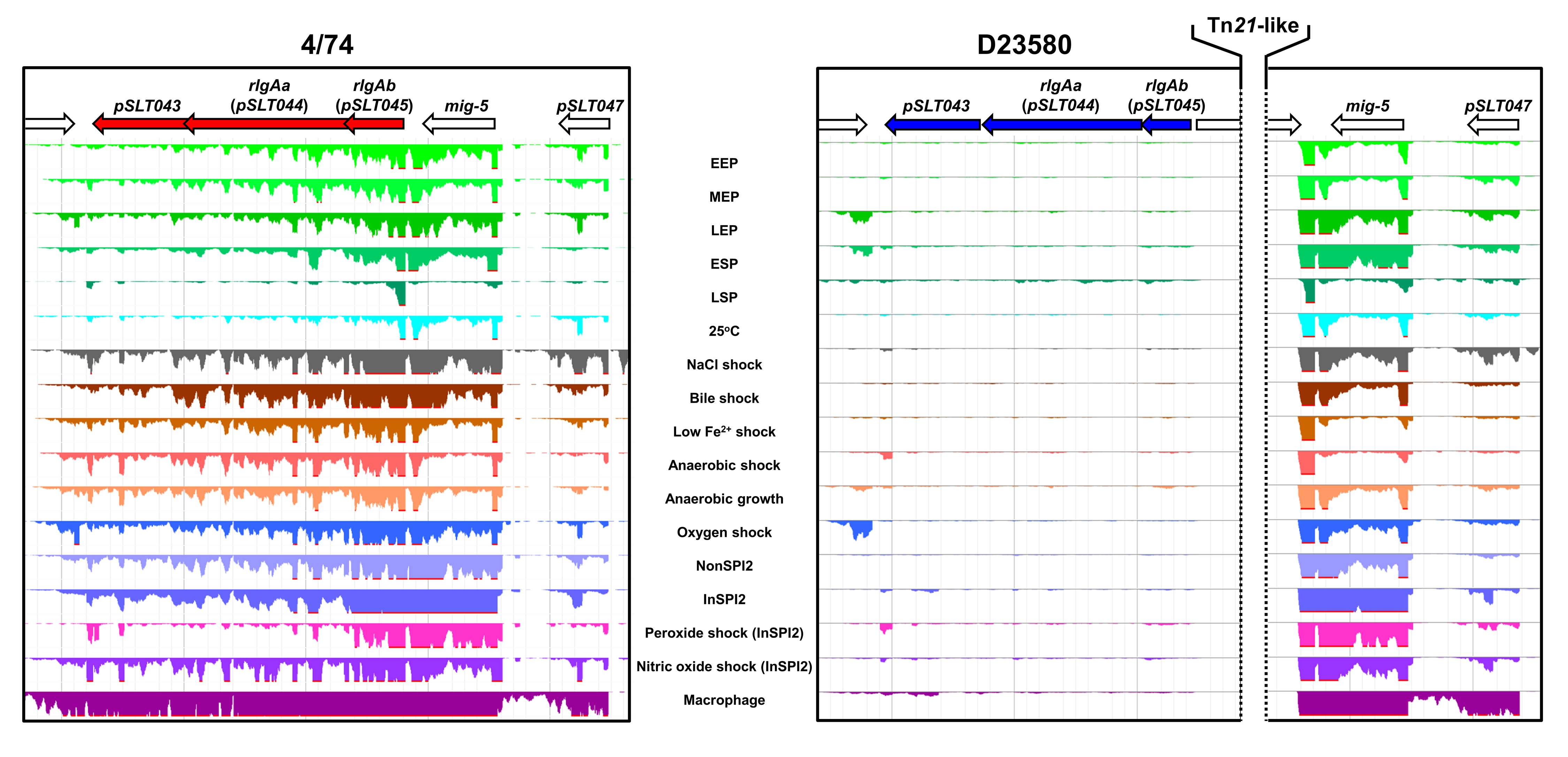

Supplement: S4 Fig — Visualization of the RNA-seq data in the 17 infection-relevant conditions from the online JBrowse resources provided in this study. Red arrows represent genes that showed up-regulation in 4/74 versus D23580, and blue arrows represent D23580-down-regulated genes. Scale of the mapped reads was 1 to 500. The insertion of the Tn21-like element is indicated by dotted lines. RNA-seq, RNA sequencing. (TIF) [file pbio.3000059.s016.tif]

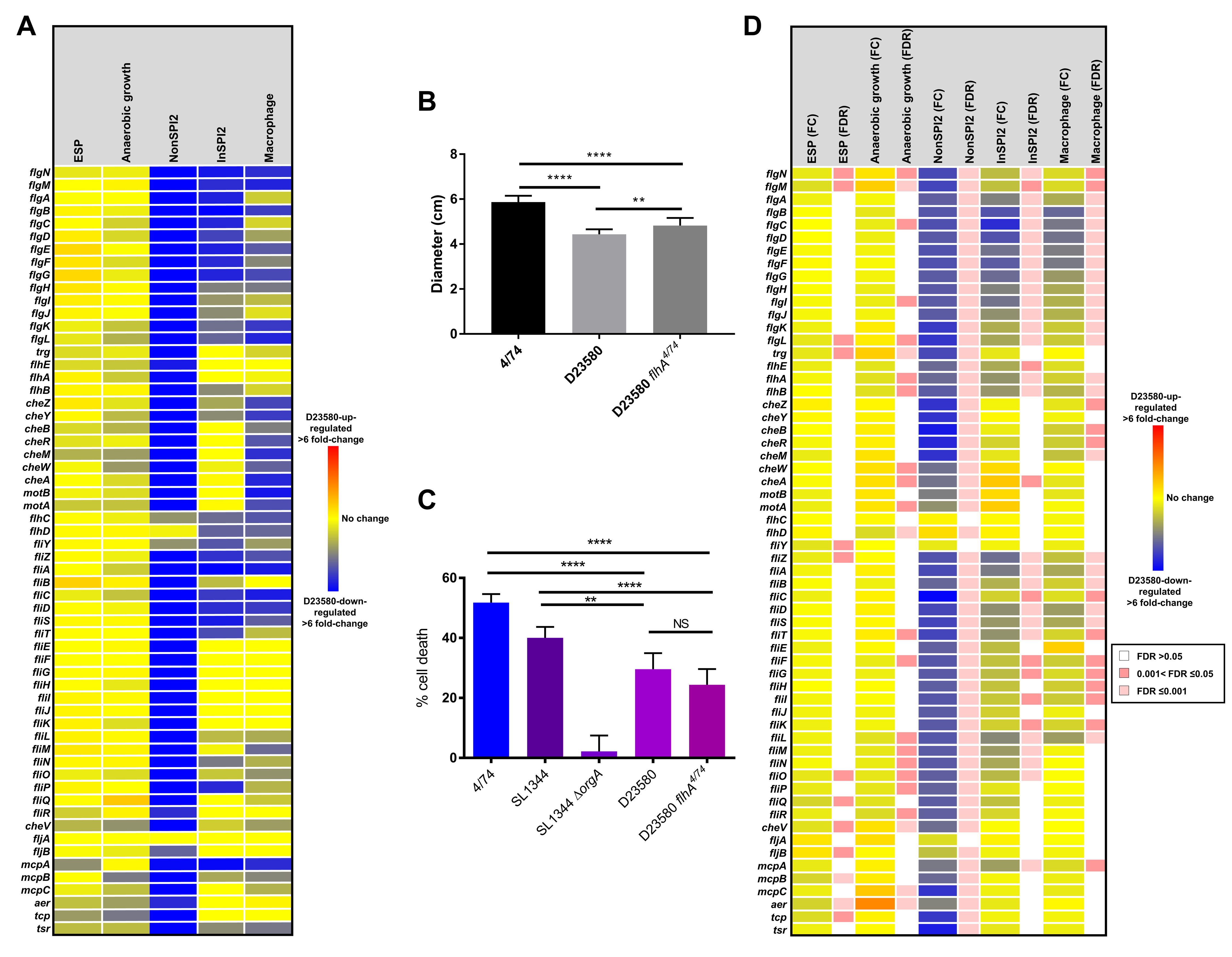

Supplement: S5 Fig — (A) Heat map of the flagellar regulon genes representing the relative expression of D23580 versus 4/74 in five growth conditions. TPM values were obtained from the RNA-seq dataset with only one biological replicate. (B) Swimming motility assay of 4/74, D23580, and D23580 flhA4/74 (S1 Text, S1 Data). Bars represent the mean of 12 independent replicates and standard deviation. Statistical analysis was determined by one-way ANOVA and Tukey’s multiple comparison test. Significant differences indicate ****, p-value <0.0001; and **, p-value <0.01. (C) LDH cytotoxicity assay using C57BL/6 BMDM (S1 Text, S1 Data). Bars represent the mean of six independent replicates and standard deviation. Groups were compared using one-way ANOVA and Tukey’s multiple comparisons test, significant differences indicate ****, p-value <0.0001; and **, p-value <0.01. (D) Heat map of the flagellar regulon using the RNA-seq data with three biological replicates. Results represent the fold-change (D23580 versus 4/74) and FDR values obtained from Degust. BMDM, bone marrow-derived macrophages; FC, fold-change; FDR, false discovery rate; LDH, lactate dehydrogenase; RNA-seq, RNA sequencing; TPM, transcripts per million. (TIF) [file pbio.3000059.s017.tif]

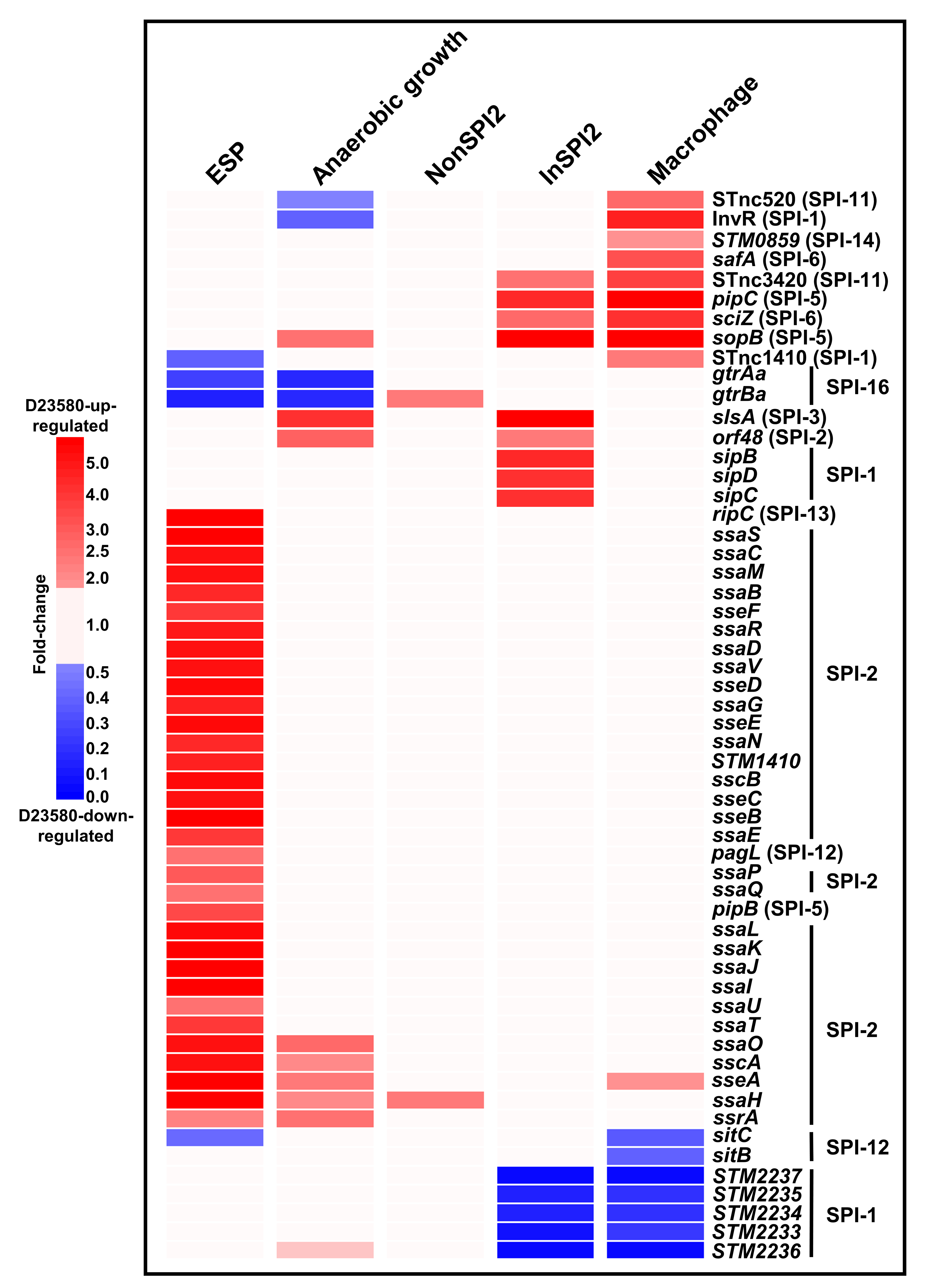

Supplement: S6 Fig — Heat map of the SPI genes and sRNAs that show ≥2 fold-change and ≤0.001 FDR (D23580 versus 4/74), obtained using GeneSpring GX7.3 (Agilent). The CPM values of three biological replicates for each coding gene and sRNA in each condition in D23580 were compared to the CPM values for the same gene/sRNA and condition in 4/74 (S6 Table). Only fold-changes ≥2 with an FDR ≤0.001 are represented in red (D23580-up-regulated) or blue (D23580-down-regulated). CPM, counts per million; FDR, false discovery rate; SPI, Salmonella pathogenicity island; sRNA, small RNA. (TIF) [file pbio.3000059.s018.tif]

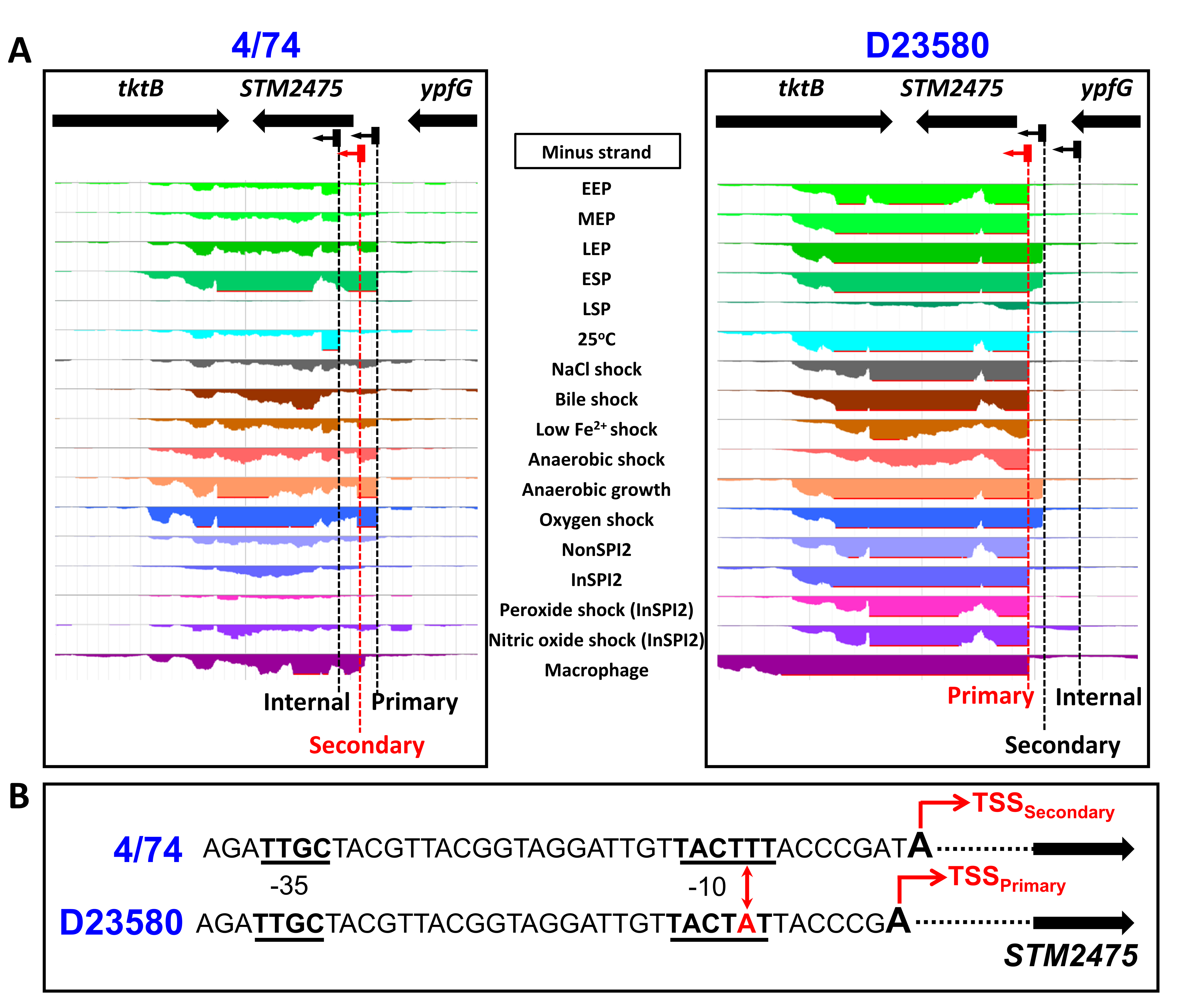

Supplement: S7 Fig — (A) RNA-seq data of 4/74 and D23580 in the 17 in vitro growth conditions for the STM2475 gene. Previously identified TSSs are indicated in both strains [20,37]. Scale of the mapped reads was 1 to 500. (B) STM2475 promoter regions of the secondary TSS in 4/74 and the corresponding primary TSS in D23580 highlight an “A” insertion in the −10 element of D23580. RNA-seq, RNA sequencing; TSS, transcriptional start site. (TIF) [file pbio.3000059.s019.tif]

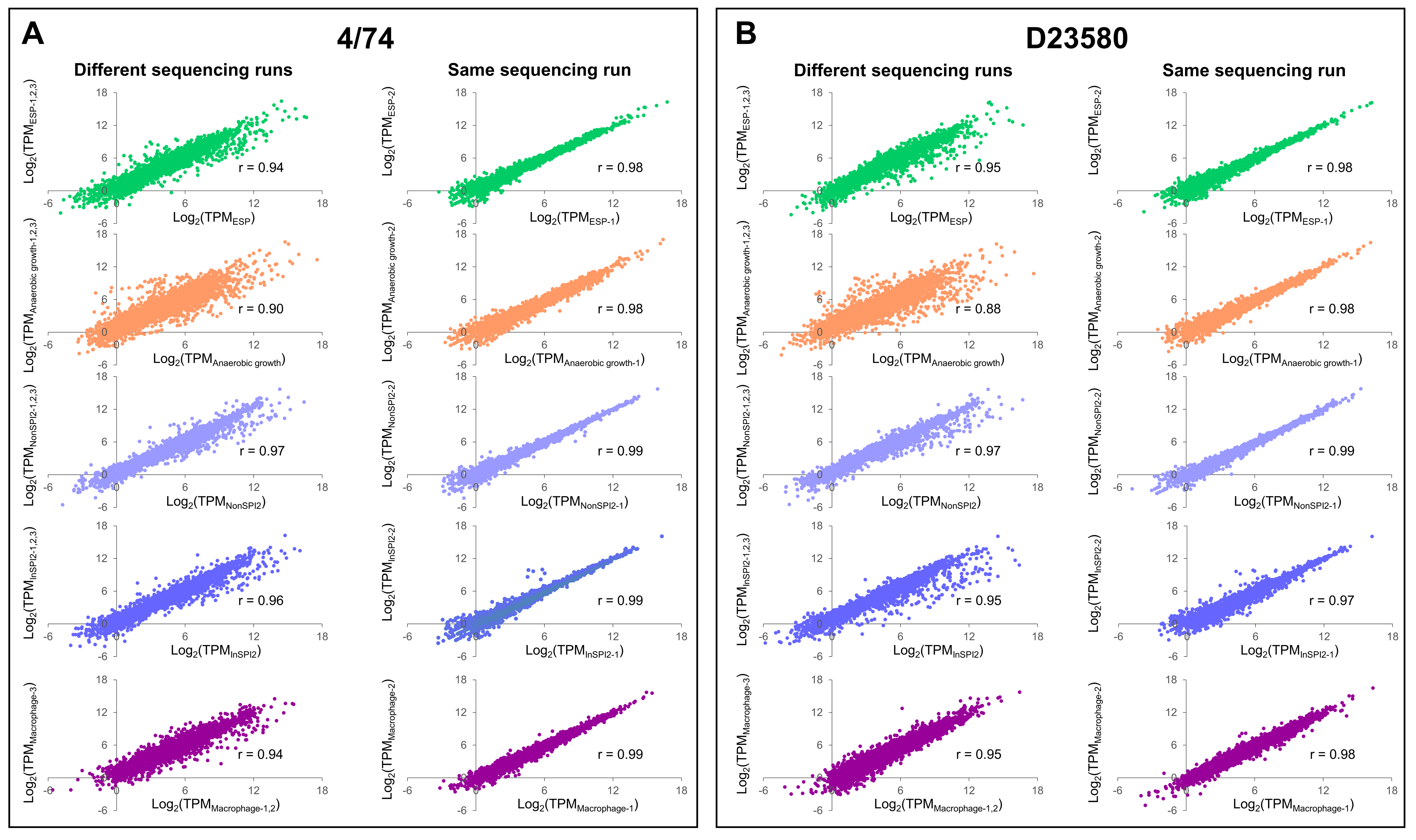

Supplement: S8 Fig — Correlation coefficient plots of Log2[TPM values] for five infection-relevant conditions. The “different sequencing runs” plots compare the two RNA-seq datasets (one biological replicate versus three biological replicates). The “same sequencing run” plots compare two samples of the RNA-seq dataset with three biological replicates. (A) 4/74, (B) D23580. RNA-seq, RNA sequencing; TPM, transcripts per million. (TIF) [file pbio.3000059.s020.tif]
